# Characterization of chromatographic yeast extract fractions promoting CHO cell growth

**DOI:** 10.1186/1753-6561-5-S8-P99

**Published:** 2011-11-22

**Authors:** Mathilde Mosser, Romain Kapel, Arnaud Aymes, Laurent-Michel Bonanno, Eric Olmos, Iris Besançon, Dominique Druaux, Isabelle Chevalot, Ivan Marc, Annie Marc

**Affiliations:** 1 Laboratoire Réactions et Génie des Procédés, UPR CNRS 3349, Nancy-Université, Vandœuvre-lès-Nancy, France; 2 Bio Springer, Maisons-Alfort, France

## Background

Many studies underline the great benefits of yeast extracts (YE), used as supplements in animal free culture media, on cell growth and recombinant protein production [1,2]. Nevertheless, their unknown composition and batch-to-batch variability of commercial YE remain constraints for industrial processes [3,4]. Consequently, the identification of bioactive YE molecules is challenging for process reliability. The main strategy to respond upon this problem is to fractionate the extract and then to characterize the fractions. So far, several fractionation processes such as ultrafiltration [5], gel filtration chromatography [6] or alcoholic precipitation [7] have been investigated. These studies suggested that the active components were mainly small molecules (< 1000 Da). But, the other physico-chemical properties of YE components have been poorly studied until now. In regards of this statement, it is proposed to get better knowledge of the YE molecules leading to an improvement of CHO cell growth, by implementing various chromatographic fractionation processes.

## Material and methods

CHO-AMW cell line producing anti-human RhD-IgG1 was provided by Dr. F. Wurm. Cell growth assays were performed in 96-well plates with 200 μL of working volume inside an incubator at 37 °C and 5 % CO_2_. Cells were cultivated in a protein-free chemically defined medium RPMI 1640/BDM (90/10) supplemented with 4 mM of glutamine (Eurobio, France) and supplemented or not by 1 g.L^-1^ of YE fraction. Cell concentration was in-situ monitored by Cellscreen^®^ analyser (Innovatis, Germany).

Raw YE, the soluble fraction of yeast autolysate, was provided by BioSpringer. A nanofiltration step using Nanomax 50 membrane (Millipore, Bedford, MA) was used to remove the very small molecules such as salts and amino acids. YE was first fractionated by three one-step preparative chromatographies according to the described conditions (Table 1). Fractions containing salts and buffers were purified and concentrated by nanofiltration. In another way, a complete chromatographic fractionation was achieved by sequentially separating YE with anion-exchange, hydrophobic interaction and size exclusion chromatography. All fractions were lyophilized for further analyses and cell culture assays.

**Table 1:** Operating conditions of preparative chromatographic fractionations.

Separation	Gel / Column	Fractions
Anion-exchange	Q Sepharose, H 10 cm, ϕ 10 cm	A.1, A.2, A.3
Hydrophobic interaction	Phenyl Sepharose , H 10 cm, ϕ 5 cm	H.1, H.2
Size-exclusion	G-15 Sephadex, H 75 cm H, ϕ 5 cm	W.1, W.2, W.3
Sequentially three step process	same than one step processes	A.x-H.y-W.z

Concentration of total amino acids, polysaccharides and nucleic acids were determined in all fractions by HPLC methods after total hydrolysis. Molecular weights were evaluated by size-exclusion HPLC [8].

## Results

**Figure 1:**
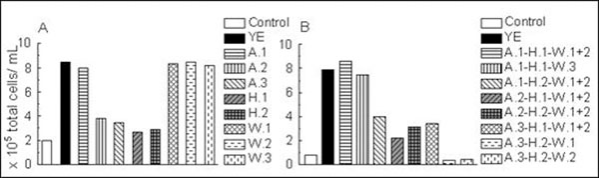
Maximal concentration of CHO cells with or without 1 g.L^-1^ of YE fractions obtained (A) by one-step and (B) three-step chromatography. The control culture was performed in the chemically-defined medium.

Firstly, the YE was separately fractionated either by anion exchange (AEC), hydrophobic interaction (HIC) or gel filtration chromatography (GFC). The AEC allowed isolating in one fraction the nucleic acids strongly bound to the gel, while peptides were distributed among all fractions depending on their electrical charges. In the HIC fractions, important quantities of buffer salts still remained despite the desalting process. Finally, the GFC led to three fractions containing various proportions of nucleic acids, polysaccharides and peptides. Then, the influence of the fractions on the CHO cell growth was evaluated in 96-well microplates (Figure 1-A). All GFC fractions presented similar results than raw YE. The HIC fractions did not stimulate the cell growth, probably due to residual buffer salts. Furthermore, only one fraction from AEC, devoid of nucleic acids but enriched in positive-charged peptides, presented a similar impact than YE.

Secondly, YE was divided in 18 fractions by a global process including the three chromatographic methods in a sequential mode: AEC, HIC, GFC. The final GF step presented the advantage to remove the residual salts. The cell growth monitoring pointed out two fractions, which led to similar results than the YE (Figure 1-B). These fractions contained peptides, mainly positively charged and polar, low quantities of polysaccharides but no nucleic acids. The amino acids quantification of fraction A.1-H.1-W.3 underlined that peptides were mainly composed of lysine and arginine. This result was consistent with a recent patent claiming that C-terminal arginine containing tripeptides were at least partially responsible for the growth promoting activity of a peptone (9).

## Conclusion

A one step-fractionation process by anion exchange chromatography led to a simplified composition of raw YE without compromising its stimulating effect on CHO cell growth. Furthermore, the three-step fractionation process allowed a better knowledge of the physico-chemical properties of the molecules involved in cell growth stimulating effects: polar positively charged and mainly composed of peptides enriched in arginine and lysine residues.
